# Recent Developments in the Diagnosis of Mucormycosis

**DOI:** 10.3390/jof8050457

**Published:** 2022-04-28

**Authors:** Eric Dannaoui

**Affiliations:** 1 Unité de Parasitologie-Mycologie, Service de Microbiologie, Hôpital Européen Georges-Pompidou, AP-HP, F-75015 Paris, France; eric.dannaoui@aphp.fr; 2 UR Dynamic 7380, UPEC, EnvA, USC ANSES, Faculté de Santé, F-94000 Créteil, France; 3 Faculté de Médecine, Université Paris Cité, F-75006 Paris, France

**Keywords:** mucormycosis, mucorales, molecular diagnosis, qPCR

## Abstract

Mucormycosis is a potentially fatal infection that presents in different clinical forms and occurs in patients with various risk factors. Recently, the COVID-19 epidemic has been responsible for an increase in the incidence of mucormycosis, particularly in India. As with other invasive filamentous fungal infections, there are no specific clinical or radiological signs, and we have fewer diagnostic tools available than for other invasive fungal infections. Therefore, the diagnosis of Mucormycosis remains difficult. Nevertheless, for optimal management, early and accurate diagnosis is important. According to the latest recommendations, diagnosis is based on direct examination of clinical specimens, and/or histopathology, and culture. There are also molecular tools for direct detection from clinical specimens, but these techniques are moderately recommended. The main problems with these molecular techniques are that, until now, they were not very well standardized; there was a great heterogeneity of DNA targets and methods, which resulted in variable sensitivity. It is in this field that most advances have been made in the last two years. Indeed, recent studies have evaluated the performance and kinetics of Mucorales qPCR in serum and have shown good sensitivity and specificity. Large inter-laboratory evaluations of qPCR in serum have also been performed and have demonstrated good qualitative and quantitative reproducibility. These new results suggest the use of Mucorales qPCR as part of the diagnostic strategy for mucormycosis. One way to achieve better reproducibility could be to use commercial methods. Currently, there are at least three commercial qPCRs for Mucorales (MucorGenius from PathoNostics, MycoGenie from Ademtech, and Fungiplex from Bruker) that can be used to test serum, respiratory samples, or biopsies. However, to date, there has been little evaluation of these methods. Overall, Mucorales PCR in tissue samples, in respiratory samples, and in serum is promising and its addition as a diagnostic tool in the definitions of invasive mucormycosis should be discussed.

## 1. Introduction

The epidemiology of mucormycosis is complex and evolving [1,2]. Over the last few years, we have seen an increasing number of cases in different parts of the world [1]. There are multiple risk factors for mucormycosis such as hematological malignancies and neutropenia, but the infections are also seen in transplant patients, diabetic patients, or following trauma or burns [2]. Interestingly, these risk factors vary across regions and countries. For example, in some countries such as Iran or India, most mucormycosis patients are diabetic patients with rhino-orbito-cerebral forms, whereas in Europe, most cases are pulmonary forms observed in immunosuppressed patients [1]. The clinical presentation depends not only on the underlying disease, but also on the species involved [1]. Many species can cause mucormycosis, and recently emerging and/or new species have been reported, such as *Saksenaea erythrospora* [3] or *Rhizopus homothallicus* [4,5].

Due to the COVID-19 pandemic, we have seen an upsurge in mucormycosis cases in the last two years, especially in India [6,7,8]. In Europe and other parts of the world, some cases of mucormycosis have been reported in patients hospitalized in ICU for severe COVID-19 [6,9,10,11]. For example, in the recently published MYCOVID study in France, six patients with mucormycosis have been reported representing about 1% of the patients with severe COVID-19 [10]. Another recent report from France also showed that mucormycosis may be a deadly complication of COVID-19 [9].

It is therefore essential to be able to make a rapid and accurate diagnosis of this infection.

## 2. Current Diagnostic Tools and Recommendations

Diagnosis of mucormycosis remains difficult and there are fewer tools available than for other invasive fungal infections.

According to current recommendations [12,13], diagnosis is based primarily on direct examination and culture, both of which are strongly recommended. Direct examination of clinical samples can be performed using special stains, such as methenamine silver stain or fluorescent brighteners. Culture is also very important because it first allows for accurate identification to the species level and then for antifungal susceptibility testing [12] which is important for a better epidemiological knowledge of these resistant fungi [14]. The main problem of these techniques is their lack of sensitivity as only 50% of cases are culture-positive [15]. For isolates obtained in culture, it has been clearly shown that molecular identification is more accurate than morphology [13]. For this purpose, ITS sequencing is the most recommended and is more accurate than other DNA targets [13]. Antifungal susceptibility testing is recommended for better epidemiological knowledge but marginally recommended for guiding treatment. Histopathology is also strongly recommended. Immunohistochemistry is possible by using monoclonal antibodies that are commercially available, but it needs trained personnel and specialized laboratories.

For direct detection in clinical specimens (fresh tissue, formalin-fixed and paraffin-embedded (FFPE) tissue, blood, or other body fluids), several molecular methods have been evaluated, but these techniques are currently moderately recommended. The main concerns with these molecular techniques were that they were not commercially available, they were not very well standardized, there is a high heterogeneity of DNA targets and methods used, and the sensitivity was variable.

This is the area where most advances have been made in the last two years. As several reviews on the diagnosis of mucormycosis have been published over the last few years [15,16,17,18,19,20], I focused on the most recent data.

## 3. Advances in Molecular Diagnosis

### 3.1. PCR in Serum

Very recently, a French prospective multicenter study (the MODIMUCOR study) which evaluates a Mucorales quantitative PCR in serum for the diagnosis of invasive mucormycosis has been published [21]. There were 245 patients divided into two cohorts. The first cohort included 232 patients with suspicion of invasive mold disease that were prospectively followed and a second cohort of additional patients with probable or proven mucormycosis were added to study the fungal load kinetics. In total, there were 40 cases of mucormycosis, 27 patients in Cohort 1, and 13 in Cohort 2. Mucorales qPCR was performed twice a week in each participating center. This qPCR, previously published [22], comprised three qPCR assays targeting *Rhizomucor* spp., *Lichtheimia* spp., and *Mucor* spp./*Rhizopus* spp., respectively.

The performance of the qPCR was first evaluated on Cohort 1, on the 27 patients. Mucormycosis were mostly pulmonary or disseminated and there was a lower number of rhino-orbito-cerebral cases. Among the 27 patients, 23 had a positive PCR in serum and 4 were negative. There were also 21 patients with a positive qPCR in the group without mucormycosis. Overall, the sensitivity was 85% the specificity about 90% with a high negative predictive value of almost 98%. The qPCR was also evaluated on the whole population of 40 mucormycosis cases. Mucorales-positive cultures were obtained for 25/40 patients. Various species were detected, *Rhizopus*, *Mucor*, *Lichtheimia*, and *Rhizomucor*. More identifications were obtained directly from the tissues by molecular identification, and 36/40 had a positive qPCR in serum. Genera detected in serum by the Mucorales qPCR were 100% in accordance with the species identified from the tissue or bronchoalveolar lavage (BAL) samples.

The first positive qPCR was observed at a median of four days before the first positive mycological or histological specimen and one day before the first imaging. Survival at 30 days and 6 months was significantly higher among patients with a qPCR becoming negative within 7 days after treatment initiation than among patients for whom the qPCR remained positive

### 3.2. Reproducibility

Until now, there were few data about reproducibility of Mucorales PCR. Recently, a large interlaboratory evaluation of qPCR in serum has been conducted [23]. There were 23 European laboratories that participated to the study, 12 laboratories from France, and 11 laboratories from the fungal PCR initiative. The authors prepared two different panels that were tested in the participating centers by four main qPCR assays (A, B, C, D).

The first panel (Panel 1) consisted in three sera that were spiked with Mucorales DNA, with three different species (*R. pusillus*, *R. oryzae*, and *L. corymbifera*). There was also a negative control included. Overall, there were 18 different protocols used depending on the laboratories (one of the methods was a commercial PCR kit). Globally, the qualitative results (positive or negative) were correct in 94–100% of cases. A low interlaboratory variability in Cq values was observed, particularly for Method A which is the method used in the MODIMUCOR study with a variability of less than two cycles. The second panel (Panel 2) consisted of six sera with three concentrations of DNA for two species (*R. pusillus* and *L. corymbifera*). The detection rate was high, ranging from 77% to 100% for the two highest concentrations, and from 50% to 85% for the lowest concentration.

The conclusion of the study is that the good reproducibility and performance support the use of Mucorales qPCR as part of the diagnostic strategy for mucormycosis.

### 3.3. Commercial Methods

One way to achieve reproducibility could be to use commercial methods. Currently, there are at least three commercial qPCR for Mucorales: The MucorGenius^®^ from PathoNostics, The MycoGenie^®^ *Aspergillus*-Mucorales species from Ademtech, and the Fungiplex^®^ from Bruker (Table 1). To date, there have been few evaluations published in the literature, except for the MucorGenius^®^ for which there are three papers [23,24,25].

These kits can be used to test serum, biopsies, including FFPE tissue samples, and respiratory specimens such as BAL. All the kits detect the most common species of Mucorales, including the most common in Europe (*Rhizopus*, *Mucor*, *Lichtheimia*, *Rhizomucor*, and *Cunninghamella*).

The MucorGenius^®^ from PathoNostics has been evaluated in three studies [23,24,25]. The first one evaluated the diagnostic and kinetic properties of the PCR in serum. In this retrospective study from Belgium, patients with invasive mucormycosis were selected based on positive cultures over a 10-year period [25]. One hundred and six blood samples from 16 patients were tested. The PCR was positive for 12 out of the 16 patients, leading to a sensitivity of 75%. Interestingly, the PCR was positive at a median of 8 days before the first positive culture, and 3 days before the first sign by imaging.

In the interlaboratory evaluation of the Mucorales qPCR mentioned above [23], among the four main qPCR assays (A, B, C, D), Method D was the MucorGenius^®^ commercial kit. When testing the six sera with different concentrations of DNA for *Rhizomucor* and *Lichtheimia*, Method A and the commercial Method D showed the highest positivity rates and the lowest Cq values.

Another study evaluated the MucorGenius^®^ kit in respiratory samples [24]. It was a retrospective study from France, including 319 patients. Among the 73 patients with proven or probable invasive mold infections, there were 10 invasive mucormycosis. All the 319 pulmonary samples (which were mainly BAL) were tested by the commercial kit and an in-house PCR. Among the 10 patients with invasive mucormycosis, 10 had an in-house positive PCR and nine were positive by the MucorGenius^®^ kit. There were also positive PCRs in the group of patients with possible mucormycosis, and few PCRs were positive in patients without invasive mold infections, and the specificity was calculated in this group. Overall, the sensitivity was 100% for the in-house PCR and 90% for MucorGenius^®^, and the specificity was over 95% for both tests.

## 4. Other Diagnostic Techniques

Besides molecular methods, other techniques—such as immunohistochemistry—have been further evaluated for the diagnosis of mucormycosis.

A recent retrospective study evaluated immunohistochemistry on FFPE tissue samples [28] by using a commercial *R. arrhizus* monoclonal antibody along with an anti-*Aspergillus* antibody. Thirteen patients with proven mucormycosis and 20 patients with invasive aspergillosis were included. Overall, the sensitivity and specificity were 100% for mucormycosis.

In another study from China, a combination of several techniques for the diagnosis of mucormycosis on FFPE tissue samples was evaluated [29]. The authors used a sophisticated method of LASER dissection prior to DNA extraction, and then used three different techniques: (i) a qPCR, (ii) a fluorescence in situ hybridization with a Mucorales-specific molecular probe, and (iii) an immunohistochemistry with a commercial anti-*Rhizopus* antibody. The study included 17 patients with mucormycosis, and the results showed that the combination of the three techniques could detect all the positive samples. Interestingly, in this study it was possible to identify *Mucor irregularis*, which is a rare Mucorales responsible for chronic cutaneous mucormycosis in Asia [30,31].

## 5. Conclusions

In the last 2 years, many advances have been made in the diagnosis of mucormycosis, in particular in the field of molecular techniques. qPCR can be used in a variety of samples (biopsies, BAL, serum). The detection of Mucorales DNA is particularly interesting in serum because it is noninvasive, it can be positive before the mycological or histopathological diagnosis, and before imaging signs. In addition, monitoring of these molecular markers could be of interest for evaluating treatment. Although some commercial qPCRs are available, further studies are needed to validate these kits.

## Figures and Tables

**Table 1:** Available commercial methods for molecular diagnosis of mucormycosis.

	MucorGenius^®^ Real-Time PCR	MycoGENIE^®^ Aspergillus Species—Mucorales Species	Fungiplex^®^ Mucorales RUO PCR Kit
Diagnostic specimens	Bronchoalveolar lavage	Serum	Not specified
	Biopsy samples, paraffin embedded	Biopsies	
	Serum	Lower respiratory tract samples	
Species detected	*Rhizopus* spp. *Mucor* spp. *Lichtheimia* spp. *Cunninghamella* spp. *Rhizomucor* spp.	*Rh. Pusillus* *M. indicus* *M.circinelloides* *M.plombeus* *R. arrhizus* *R. stolonifera* *L. corymbifera* *L. glauca* *C. bertholletiae* *Mycotypha* sp.	*Rhizopus* spp. *Lichtheimia* spp. *Cunninghamella* spp. *Rhizomucor* spp. *Mucor* spp. *Actinomucor* spp. *Apophysomyces* spp. *Saksenaea* spp. *Syncephalastrum* spp.
Manufacturer	PathoNostics	Ademtech	Bruker
Reference	[23,24,25]	[26]	[27]

## Data Availability

Not applicable.
